# Eighteen-Year Follow-up After Autologous Chondrocyte Implantation on Medial and Lateral Femoral Condyles: A Case Report

**DOI:** 10.7759/cureus.62913

**Published:** 2024-06-22

**Authors:** Austin M Paradis, Scott D Gillogly

**Affiliations:** 1 School of Medicine, University of South Carolina, Columbia, USA; 2 Orthopaedic Surgery, Georgia Bone and Joint Surgeons, P.C., Atlanta, USA

**Keywords:** orthopedic sports, orthopedic surgery, femoral condyle, medial condyle, autologous chondrocyte implantation, articular cartilage defect

## Abstract

Articular cartilage defects are common injuries of the knee. The defects often progress in size and produce significant clinical symptoms due to the lack of intrinsic repair or regenerative capacity of articular cartilage. With the failure of nonoperative treatment options, surgical treatment is indicated and includes palliative, reparative, and regenerative options. For large defects of the femoral condyles, trochlea, or patella, autologous chondrocyte implantation can provide successful and long-lasting results.

Presented is the case of a 37-year-old male with an 18-year follow-up to autologous chondrocyte implantation for extensive left knee articular cartilage defects of the medial and lateral femoral condyles. Recovery from articular cartilage defects is shown through both clinical improvement of the patient and arthroscopic photographs of robust autologous articular cartilage on the medial femoral condyle. This case supports the long-term benefits of autologous chondrocyte implantation as a surgical intervention for large, full-thickness articular cartilage defects of the knee.

## Introduction

Articular cartilage defects in the knee can produce pain and disability symptoms to the same extent as advanced osteoarthritis or anterior cruciate ligament (ACL) deficiencies [1]. Such deficits greatly impair athletic performance and quality of life parameters. Cartilage defects are known to expand in size over time causing further degradation of articular cartilage [2]. Therefore, earlier detection and definitive treatment can provide symptom relief and improved function while delaying or preventing progression to osteoarthritis [3].

This case report documents a 37-year-old male with an 18-year follow-up after autologous chondrocyte implantation (ACI) on extensive left knee defects of the medial and lateral femoral condyles. This case further supports the long-term benefits of ACI as a surgical intervention for large full thickness articular cartilage defects in the knee. Autologous chondrocyte implantation has been shown to be an effective and durable solution for the treatment of large full-thickness cartilage lesions of the knee joint. Studies support that the clinical and functional outcomes remain high even 10 to 20 years after the implantation [4].

## Case presentation

**Figure 1:**
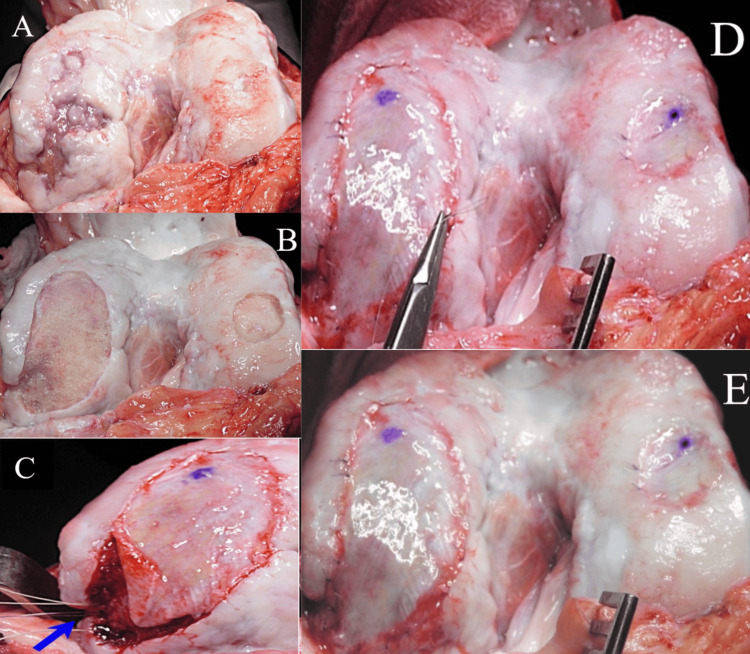
Intraoperative photographs of the patient’s ACL in 2003 The medial and lateral condylar defects shown are undebrided (A) and then debrided (B). Anchors for the uncontained area were placed (C) and the periosteum was fully sutured in place with interrupted 6-0 Vicryl sutures (D). The final repair after the suture line is sealed with fibrin glue and the autologous cells injected below the secured periosteal membrane is shown (E). ACL: anterior cruciate ligament

A 37-year-old male presented for an evaluation of a complex left knee problem dating back to a high school sports injury in 2001. At an outside institution, he had undergone an ACL reconstruction followed by an indolent intra-articular infection, debrided, and treated with long-term antibiotics. He later underwent a revision ACL reconstruction at another institution, at which time the large cartilage defects were noted and a cartilage biopsy was obtained. He was then referred to our center in early 2003 for treatment of his cartilage defects with ACI. Intraoperative photographs of the patient’s ACI in 2003 were taken (Figures 1A-1E). At the time of surgery, he underwent first-generation ACL of a 59 mm x 29 mm medial femoral condyle defect and a 28 mm x 18 mm lateral femoral condyle defect, and concomitant anteromedialization of the tibial tubercle and hardware removal of an ACL screw. Postoperatively the patient was treated with a continuous passive motion (CPM) machine (for six hours per day), quad sets, straight leg raises, passive range of motion, and partial weight bearing for four weeks. He was then allowed to advance motion, core, pelvic, and femoral strengthening, and weight bearing as tolerated. At one year postoperatively, he was allowed to advance to full activity although he voluntarily “retired” from cutting sports (Figures 1A-1E).

In August 2020, the patient was evaluated for complaints of progressive pain in the left knee over the past year. He described the pain to occur on attempted squatting or kneeling. He also complained of hardware pain around his tibial tubercle and said that the screws are palpable and bothered him with physical activity. Examination demonstrated a 1+ effusion, palpable loose body, motion from full extension to 110 degrees, trace Lachman, negative pivot, and negative anterior and posterior drawer testing. He was neurovascularly intact and had a good quadriceps tone. There was tenderness to palpation over the prominent screws about the tibial tubercle.

**Figure 2:**
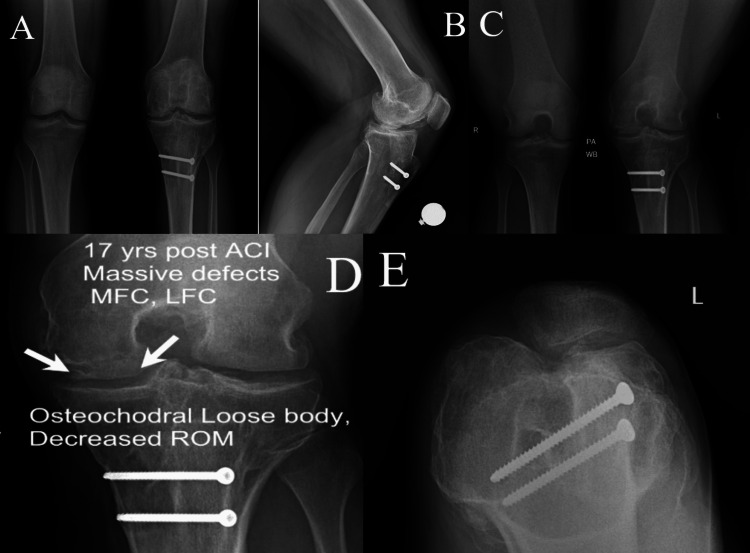
Anterior-posterior (A), lateral (B), posterior-anterior (C +D), and sunrise (E) radiographs of the patient’s knees upon initial examination; panel D is increased magnification of panel C with text superimposed to highlight the presence of an osteochondral loose body

Radiographs of both knees were obtained and demonstrated the presence of large loose bodies with preservation of the medial and lateral joint spaces. There was a questionable intra-lesional osteophyte of the trochlea on a sunrise view (Figures 2A-2E). Based on the patient’s symptoms correlating with radiographs and examination, arthroscopic removal of loose bodies, debridement, and removal of retained hardware was recommended.

**Figure 3:**
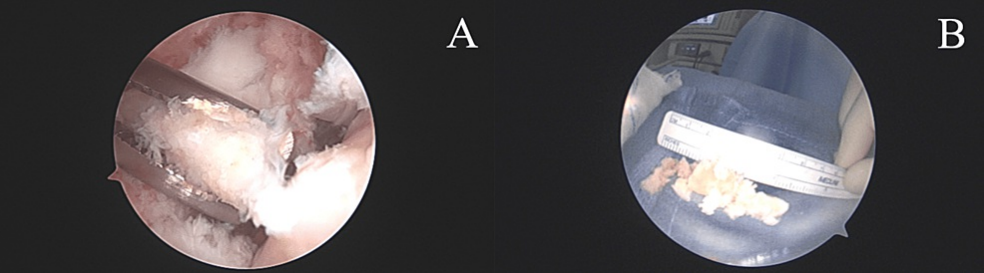
Intraoperative, arthroscopic photos of removal of osteochondral loose bodies (A) and the accumulated size of the loose bodies removed (B).

**Figure 4:**
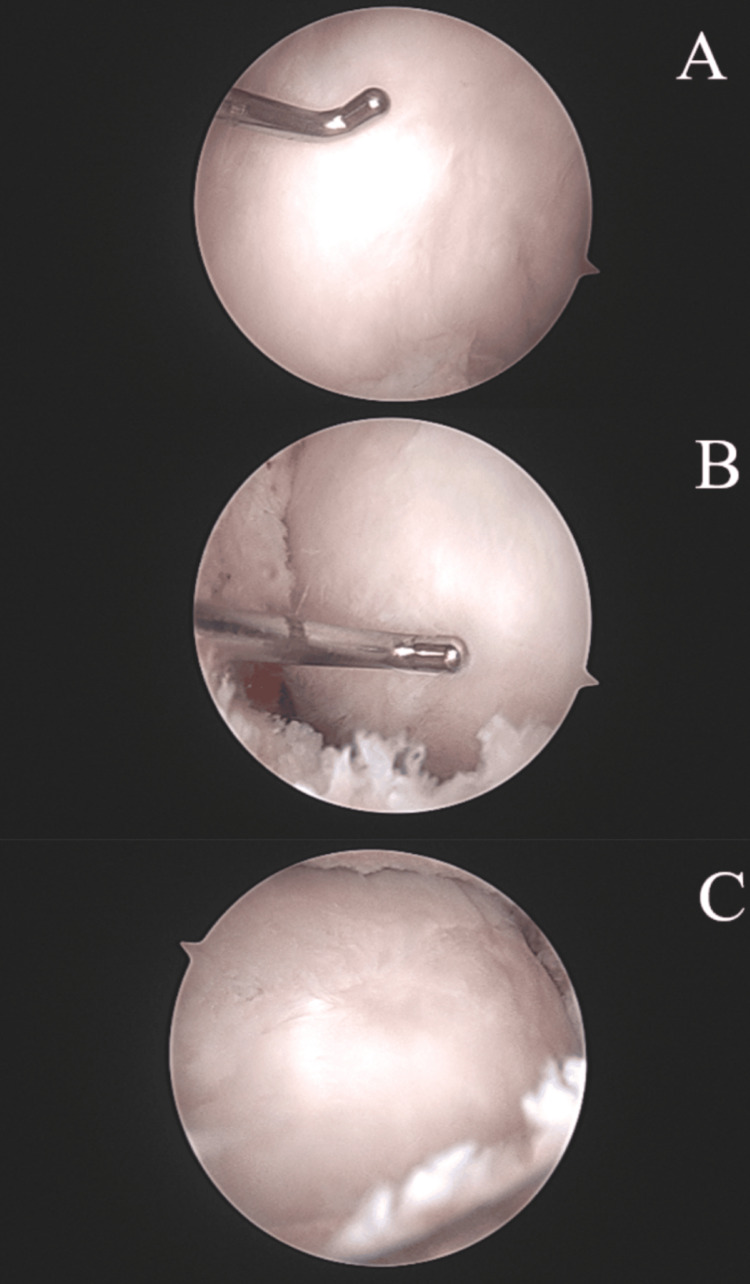
Intraoperative, arthroscopic photos (A+B+C) of the medial femoral condyle 17 years post autologous chondrocyte implantation

The patient then underwent the recommended procedure with an exam under anesthesia unchanged from the preoperative exam. In addition to standard anterolateral and anteromedial portals, superomedial and superolateral portals were made to assist with the lysis of extensive adhesions and the removal of large loose bodies that were partially embedded in scar tissue in the anterior and patellofemoral compartments. The loose body in the anterior compartment was greater than three centimeters in size. The loose bodies were fragmented and resected from the scar tissue so that they could be removed through the portals (Figures 3A-3B). After lysis of the adhesions and removal of the osteochondral loose bodies, there was full visualization of the femoral condyles. The medial femoral condyle showed robust smooth articular cartilage with appropriate firmness with the ACL site fully repaired. The lateral femoral condyle showed intact repair tissue with a new area of chondral involvement in the lateral trochlea, which was debrided, and the chondral flap was stabilized via chondroplasty (Figure 4A-4C).

The patient’s passive range of motion was improved to 120 degrees of flexion. The patient’s retained hardware was then removed from the tibial tubercle through small incisions. The wounds were all closed with absorbable sutures. Postoperatively, the patient was treated with calf pumping exercises, quad sets, passive range of motion, and weight bearing as tolerated for three weeks and progressed to femoral strengthening and return to full activity as tolerated. At four weeks postoperatively, the patient showed healed incisions, full extension, flexion to 115 degrees, good stability, decreased crepitus, and improved comfort.

**Figure 5:**
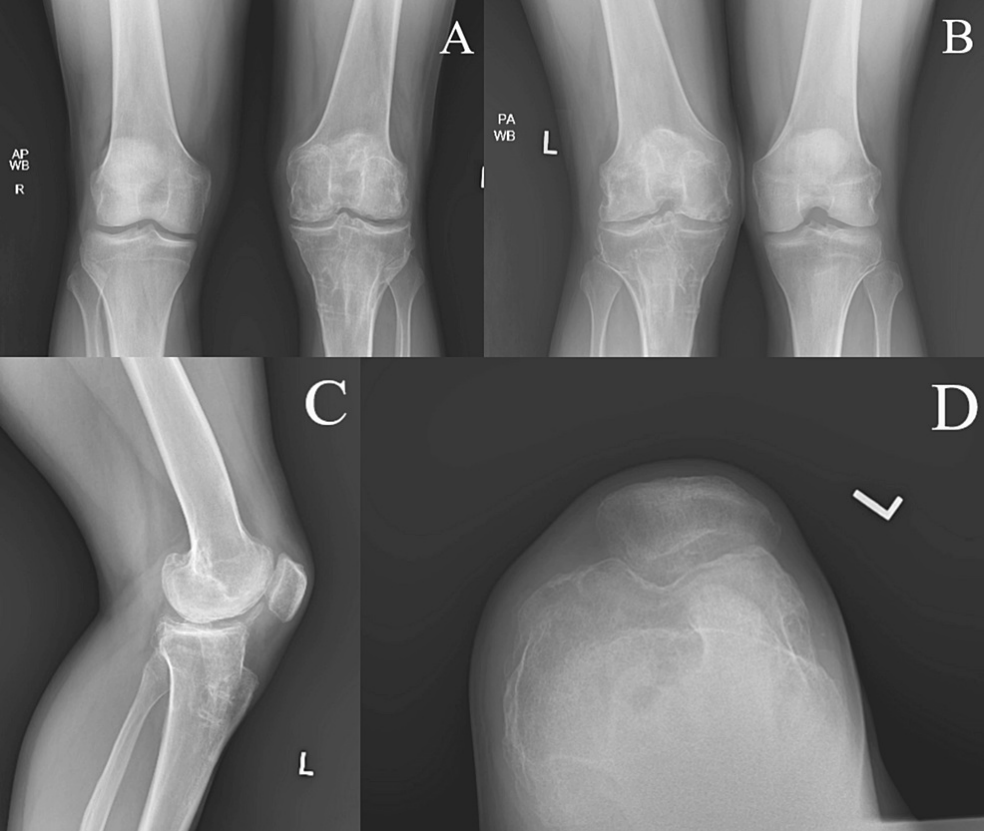
Anterior-posterior (A), posterior-anterior (B), lateral (C), and sunrise (D) radiographs of the patient's left knee one year post arthroscopic removal of loose bodies with lysis of adhesions, partial synovectomy, and hardware removal. Radiographs were also 18 years post autologous chondrocyte implantation. AP and PA showed a preserved joint space with mild subchondral irregularity.

He was again re-evaluated 15 months post arthroscopic removal of loose bodies with lysis of adhesion and partial synovectomy and 18 years post autologous chondrocyte implantation of the medial and lateral femoral condyles. Examination showed full extension and flexion to 120 degrees with no effusion. There was good stability on varus and valgus testing, as well as anterior and posterior drawer testing, and no significant pain during open chain terminal knee extension. Radiographs showed a preserved medial joint space narrowed by 25% with mild persistent subchondral irregularity (Figures 5A-5D). The patient stated that he was able to participate in cardiovascular fitness workouts and training such as running. He was able to participate as tolerated in physical activities with no restrictions.

## Discussion

An articular cartilage defect involves an injury to the cartilage that, in turn, causes disruption of proteoglycan synthesis, increased tissue hydration, and collagen disorganization. These characteristics lead to a progression of the cartilage injury, which causes a degenerative cycle of the cartilage in the joint. An articular cartilage defect can lead to pain, impaired function, joint catching/clicking, recurrent effusions, and swelling. If left untreated, the defect may lead to osteoarthritis [5-10]. Non-operative treatments that may relieve the symptoms of the defect include physical therapy, non-steroidal anti-inflammatory medication, and injection therapy. If non-operative treatment strategies fail to provide pain relief, surgical treatment strategies should be considered [11].

Articular cartilage injuries of the knee are common. Studies of over 30,000 knee arthroscopies have found that 63% of the arthroscopies revealed a cartilaginous lesion [12]. The average age of these patients was 43 years old, with ages ranging up to 92 years old. Of the arthroscopies that showed a cartilage lesion, almost 20% had a full thickness lesion with exposed bone. Articular cartilage defects affect a wide range of patients. Many of these patients are young and could benefit from a treatment option such as ACI.

Articular cartilage full thickness injuries are also prevalent in young active populations. One study evaluated the incidence and risk factors of full thickness chondral defects in college football athletes invited to the National Football League Scouting Combine over a three-year period [13]. The study found that 20.3% (198/975) of players had at least one full-thickness chondral defect detected by MRI. The study also found that 8.06% (79/975) had more than one defect. Treatment of this younger population provides additional complications because prostheses used in total knee replacement have a limited lifetime [14]. The use of autologous cellular material provides an opportunity for new cartilage to form that could potentially have a lasting effect to prevent the need for total knee replacement in young patient populations [15].

There is no single physical finding on examination specifically related to articular cartilage defects. Small or large effusions may be present. The motion of the knee is typically preserved, however, osteochondral fragments, a common complication of articular cartilage defects, can cause intermittent locking of the knee. Larger defects can cause repeatable catching or clicking during knee motion examination and patellar manipulation. Radiographs are important for evaluating for any malalignment and degenerative changes of the knee that may be related to the defect [11]. In addition to lateral and sunrise views, weight-bearing standing anteroposterior (AP) and 45-degree posteroanterior (PA) view radiographs are essential to evaluate chondral defects. Long-limb full-length hip-to-ankle anteroposterior views can be critical for the evaluation of knee malalignment [16].

Due to its avascular nature, articular cartilage has a low metabolic rate and a very poor regenerative capacity [17]. Therefore, articular cartilage defects typically do not heal without surgical intervention [3]. The size of the defect plays an integral role in the selection of the surgical treatment option. Previous studies have shown that there are different outcomes of the surgical procedure based on the size of the defect [18-21]. Small defects (<2-4 cm^2^) are treated most effectively with microfracture, marrow stimulation, osteochondral autograft transfer (OATS; Arthrex, Naples, FL), and edge stabilization procedures. Large defects (>2-4 cm^2^) are treated most effectively with ACL and osteochondral allograft transfer (OATS). Large defects are also typically found with concomitant pathology such as multiple defects, uncontained defects, and bone deficiency. In the presence of treatment of a chondral injury, concomitant knee pathology, such as malalignment, ligamentous instability, or meniscal deficiency, must also be addressed to improve the intraarticular environment for any cartilage repair process [3,11,22-24].

The ACL procedure has developed through multiple generations. The first generation involved the implantation of cultured autologous chondrocytes beneath a previously harvested periosteum sutured the edges of the chondral defect. This first-generation treatment, also known as PACL, had complications with periosteal graft detachment and the risk of subsequent procedures due to periosteal hypertrophy. The second generation of ACI involved the implantation of cultured autologous chondrocytes beneath an absorbable collagen membrane. This generation found a lower rate of graft hypertrophy compared to the first generation [25,26]. Further studies also found that more patients who received the second-generation ACL reported good to excellent results two years after their procedure compared to patients who received the first generation (82% compared to 55.5%) [27]. The third generation of ACL, also known as matrix-induced ACI or MACI, uses cultured autologous chondrocytes that are pre-seeded into a collagen membrane. This technique is less invasive and requires a shorter surgical time while still being an effective treatment for cartilage defects. A randomized study of over 140 patients treated with MACI versus microfracture for symptomatic chondral defects showed a significant improvement in 87.5% of MACI patients versus 68% of microfracture patients [28].

## Conclusions

This case study reports the excellent long-term functional and clinical outcomes for extensive chondral defects of the medial and lateral femoral condyles treated with first-generation ACL in a then-20-year-old athlete with an 18-year follow-up. Full thickness chondral defects that are symptomatic should be considered for surgical intervention. Large defects and multiple defects are both indications for treatment with autologous chondrocytes, now with the third-generation matrix autologous chondrocyte implantation (MACI). The use of ACL in this case yielded articular cartilage regeneration that proved durable and provided pain relief and markedly improved clinical outcome for the patient at 18 years post the index procedure.
